# Comprehensive analysis of SARS-CoV-2 Spike evolution: epitope classification and immune escape prediction

**DOI:** 10.1093/ve/veaf027

**Published:** 2025-06-11

**Authors:** Natália Fagundes Borges Teruel, Matthew Crown, Ricardo Rajsbaum, Matthew Bashton, Rafael Najmanovich

**Affiliations:** Department of Pharmacology and Physiology, Faculty of Medicine, Université de Montréal, Montreal, Canada; Hub for Biotechnology in the Built Environment, Department of Applied Sciences, Faculty of Health and Life Sciences, Northumbria University, Newcastle upon Tyne NE1 8ST, United Kingdom; Center for Virus-Host-Innate-Immunity, RBHS Institute for Infectious and Inflammatory Diseases, and Department of Medicine, New Jersey Medical School, Rutgers University, 205 S. Orange Avenue, Newark, NJ 07103, United States; Hub for Biotechnology in the Built Environment, Department of Applied Sciences, Faculty of Health and Life Sciences, Northumbria University, Newcastle upon Tyne NE1 8ST, United Kingdom; Department of Pharmacology and Physiology, Faculty of Medicine, Université de Montréal, Montreal, Canada

**Keywords:** SARS-CoV-2, Spike protein, structural biology, epitopes, immune recognition, conformational dynamics

## Abstract

The evolution of severe acute respiratory syndrome coronavirus 2 (SARS-CoV-2), the virus responsible for the COVID-19 pandemic, has produced unprecedented numbers of structures of the Spike protein. In this study, we present a comprehensive analysis of 1560 published structures, covering most major variants that emerged throughout the pandemic, diverse heteromerization, and interacting complexes. Using interaction-energy-informed geometric clustering, we identify 14 structurally distinct epitopes based on their conformational specificity, shared interface with angiotensin-converting enzyme 2 (ACE2), and glycosylation patterns. Our per-residue interaction evaluations accurately predict antibody recognition sites and correlate strongly with deep mutational scanning data, enabling immune escape predictions for future variants. To complement this structural analysis, we integrate longitudinal genomic data from nearly 3 million viral sequences, linking mutational patterns to changes in Spike’s conformational dynamics. Our findings reveal two distinct evolutionary trade-offs driving immune escape. First, we confirm an enthalpic trade-off, where mutations in the receptor-binding motif (RBM) enhance immune escape at the cost of weakened ACE2 binding. Second, we introduce an entropic trade-off, showing that mutations outside the RBM modulate Spike’s conformational equilibrium, reducing open-state occupancy to evade immune detection—without directly altering the ACE2-binding interface. With these analyses, this work not only highlights the different functional effects of mutations across SARS-CoV-2 Spike variants but also reveals the complex interplay of evolutionary forces shaping the evolution of the SARS-CoV-2 Spike protein over the course of the pandemic.

## 1. Introduction

The severe acute respiratory syndrome coronavirus 2 (SARS-CoV-2) virus, responsible for the global COVID-19 pandemic (Wu et al. 2020, Lu et al. 2020, Ou et al. 2020), has been the subject of extensive scientific scrutiny, particularly regarding its Spike protein, which plays a critical role in viral entry into host cells. The ability of the SARS-CoV-2 Spike protein (referred to simply as Spike from here on) to bind to the angiotensin-converting enzyme 2 (ACE2) receptor (Li, et al., 20, Ou et al. 2020), its immune recognition (Walls et al. 2020), and its glycan shield (Watanabe et al. 2020) have made it a focal point of research, leading to an unprecedented amount of structural data (Xu et al. 2023). This wealth of information has enabled researchers to study the molecular mechanisms governing viral infectivity and immune evasion, using both experimental and computational approaches.

While computational and experimental studies have provided detailed insights into specific aspects of the Spike protein related to binding (Jawad et al. 2021, Sergeeva et al. 2023) and dynamics (Verkhivker 2020, Mansbach et al., 2021, Teruel et al. 2021b, Calvaresi et al. 2023), most studies often do so in isolation, focusing on individual aspects rather than the broader functional context. Conversely, epidemiological studies tend to capture the overall effects of these mechanisms on viral spread, yet lack the granularity needed to disentangle the contributions of multiple mechanisms to the observed outcomes.

The central role of the Spike protein in mediating viral entry also makes it a prime target for immune recognition and therapeutic intervention (Walls et al. 2020). Its surface exposure and dynamic conformational changes create opportunities for immune recognition but also pose challenges to the immune system due to the presence of protective mechanisms like glycan shielding and conformational masking. Key regions, such as the receptor-binding domain (RBD) and the receptor-binding motif (RBM), are critical for neutralizing antibody responses and are often subject to evolutionary pressures that balance receptor-binding efficiency with immune evasion (Harvey et al. 2021). Dissecting how these regions evolve under such pressures provides valuable insights into the interplay between viral infectivity and immune escape.

A crucial step in understanding this interplay involves the characterization of epitopes. Beyond their role in antibody recognition, epitopes are defined by their accessibility, which is influenced by glycan shielding and conformational flexibility, as well as by the shared binding interface with ACE2 and the constraints to the mutational landscape that this necessary interaction imposes. Numerous studies have employed different experimental and computational methodologies to identify epitopes (Piccoli et al. 2020, Shrock et al. 2020, Barnes et al. 2020a, 2020b, Deshpande et al. 2021, Hastie et al. 2021, Yuan et al. 2021, Garrett et al. 2022, Chen et al. 2023, N’Guessan et al. 2023, Sankhala et al. 2024, Yisimayi et al. 2024) ranging from structure-based evaluations, such as buried surface area calculations (Deshpande et al. 2021, Yuan et al. 2021), to sequence-based enrichment analysis for epitope mapping (N’Guessan et al. 2023), and with a predominant focus on the RBD (Piccoli et al. 2020, Barnes et al. 2020a, 2020b HERE, Deshpande et al. 2021, Hastie et al. 2021, Yuan et al. 2021, Sankhala et al. 2024, Yisimayi et al. 2024). However, while experimental methodologies are often constrained by their high cost, time-consuming nature, and complexity—making frequent updates challenging—many studies based on structural analyses were limited by the availability of structural data at the time and the absence of reliable high-throughput methods for evaluations of the energetics of molecular interactions.

Computational approaches are essential for understanding viral evolution, both in the unification and evaluation of viral genetic data (Elbe and Buckland-Merrett 2017, Shu and McCauley 2017, Hadfield et al. 2018, Marjanovic et al. 2022) and, in the context of structural analysis, in characterizing specific mechanisms and exploring potential mutations and their effects (Jawad et al. 2021, Mansbach et al. 2021, Teruel et al. 2021b, Bozdaganyan et al. 2022). Such computational approaches often employ modelling techniques to introduce single or multiple mutations, investigate their functional effects, and explore the dynamic range of motion and accessibility across different conformational states (Teruel et al. 2021b, Sergeeva et al. 2023). However, these methods inherently introduce biases due to limitations in the modelling process (Saldan˜o et al. 2022, Hameduh et al. 2023), affecting subsequent analyses (Sirin et al. 2016, Teruel et al. 2023). Studies based on experimental structures—that are also subject to biases (Gao et al. 2023)—often provide a limited view of functional aspects, as they typically rely on a single or few structures (Hong et al. 2022, Mannar et al. 2022, McCallum et al. 2022) that fail to capture the full variability and dynamic nature of a moving protein, a limitation that can be seen when comparing large numbers of observations. Meanwhile, a vast amount of experimentally solved structural data remains underutilized, primarily due to limitations of computationally expensive approaches in evaluating extensive datasets and conducting detailed per-residue analyses (Sergeeva et al. 2023). By employing methodologies suitable to high-throughput applications, we can leverage this extensive structural data for more comprehensive and accurate assessments, averaging out experimental biases inherent in protein structure determination.

In this study, we perform a comprehensive structural analysis of 1560 Spike protein structures with respect to interactions with antibodies and the receptor ACE2, conformational dynamics and glycan coating. We aim to provide a broad understanding of how various structural features contribute to the function and evolution of the Spike protein. To do so, we introduce a data-driven epitope classification system and a method for mapping other complexes into one of the 14 epitopes identified within this study. Uniquely, our methodology allows us to approximate the enthalpy of pairwise-interacting complexes as the sum of pairwise residue-level interactions in a high-throughput fashion and predict the experimentally determined immune escape potential of individual mutations. Furthermore, we integrate these evaluations with longitudinal sequence analyses, enabling us to track how structural and dynamical changes have shaped immune escape over the course of the pandemic. While prior studies have suggested a trade-off between antibody escape and ACE2 binding, we formalize this enthalpic trade-off hypothesis and extend it by introducing a second, entropic trade-off hypothesis, demonstrating that mutations outside the RBM contribute to immune escape by modulating conformational dynamics rather than direct binding interfaces. Our approach provides an accessible framework to simultaneously evaluate antibody recognition, receptor binding, and conformational flexibility, revealing epistatic effects that influence mutation-driven adaptation. This work presents a comprehensive, integrative perspective on the structural evolution of the SARS-CoV-2 Spike protein, offering both mechanistic insights and a computational toolkit for evaluating future variants.

## 2. Methods

### 2.1 Structure selection and modelling

We utilized all Spike protein structures available in the RCSB Protein Data Bank until 2 October 2023. These structures were annotated based on their complexes—either with antibodies, with the receptor ACE2, or unbound—as well as based on the presence of glycans, the variant they represent, and, in the case of full homotrimeric structures, the conformational state of each Spike monomer. We utilized pairs of structures that had the exact same sequence, were published together, and represented the closed state and the one-RBD-open state to estimate open-state occupancy as described in Teruel et al. (2021a) For these pairs of structures, we rebuilt the missing loops using Modeller (Šali and Blundell 1993). The open-state estimation of occupancy is calculated only for variants for which the required structures above are present in the PDB.

### 2.2 Evaluation of interaction energies

We used the software Surfaces (Teruel et al. 2023) to evaluate the interaction free energy of complexes of Spike and antibodies, Spike and ACE2, or the interactions of glycans present in the structures. Surfaces was previously shown to predict the effects of Spike mutations on the binding affinity to the receptor ACE2 as accurately as current state-of-the-art methods based on molecular dynamics simulations at a fraction of the required computational time (Teruel et al. 2023). Surfaces generates a matrix of pairwise pseudo-energetic contributions to the enthalpy of binding. We collapsed the matrix of pairwise interactions into a vector representing the sum of all interaction enthalpies for every Spike residue. In the case of interactions with more than one chain of Spike, we focus on the interacting chain that contributes the most to the total enthalpy. We then aligned the vectors to a reference sequence in order to correct incorrectly numbered residues or adjust the numbering in cases of structures with insertions and deletions. For both per-residue evaluations and full interface interactions, we refer to the pseudo-energy calculated by Surfaces as binding affinity, as it is scaled to represent the free energy of binding in units of kcal/mol.

### 2.3 Antibody clustering

Given a protein–protein interaction interface of a Spike–antibody complex, we define a reference point as the geometric average position of all interacting residues, weighted by their contribution to the overall interface enthalpy as calculated by Surfaces:


(1) $$\left( {X,\,Y,\,Z} \right) = \frac{{\mathop \sum \nolimits_{k = 1}^n \left( {{x_k},{y_k},{z_k}} \right) \times \,{w_k}}}{{\mathop \sum \nolimits_{k = 1}^n {w_k}}}$$

where (*X, Y, Z*) represent the coordinates of the reference point, (*x_i_, y_i_, z_i_*) are the coordinates of each residue within the interface, with its corresponding contribution to the enthalpy *w; n* represents the number of interface residues.

Hierarchical clustering based on the pairwise Euclidean distance of these average points was used to cluster the 2032 antibody interactions from 942 structures of Spike–antibody complexes (Supplementary Fig. S1). This is, to our knowledge, the first instance of using interaction energies to bias geometric clustering, adjusting the relevance of the interaction interface based on the importance of the per-residue contacts. This approach resulted in 14 clusters, represented by their respective weighted average points, that are biased towards the positions with the strongest interactions in each epitope. We applied principal component analysis (PCA) to visualize the clustering results. This enabled us to project the points into a 2D space based on their weighted average interactions, simplifying the interpretation of the clusters.

### 2.4 Vector sorting

We calculate the distance of the average points of 2032 antibody interactions, as described by Equation (1), to each of the 14 reference points representing the characterized epitopes. This method provides greater clarity than considering all pairwise distances, especially in differentiating clusters that are very close in space, such as epitopes 4 and 5.

Employing the same strategy described by Equation (1), the 209 ACE2–Spike interaction interfaces, based on 146 complex structures, are mapped to unique epitopes by finding the epitope reference point closest to the average point of interaction for the particular ACE2–Spike interface.

The equation above is also used to analyse single residues, particularly residues in positions that undergo conformational changes or glycosylated residues. In the case of single residues, its reference point as calculated by the equation above reduces to the coordinates of the given residue atoms—and likewise for entire interfaces, this reference point is mapped to the epitope closest in space.

### 2.5 Occupancy evaluation

We employed the NRGTEN as described by Mailhot and Najmanovich (2021) to evaluate the occupancy of conformational states of the Spike protein. This method builds upon previous work on the structural dynamics of the Spike protein (Teruel et al. 2021a), incorporating Markov Chain modelling to calculate conformational state occupancies.

In the Markov model, each conformation of the Spike protein is represented as a state, with transition probabilities between states calculated using the ENCoM model. A constant *k* is added to all states, representing the probability of remaining in that state. To ensure that the transition probabilities from each state sum to 1, these values are normalized after the addition of *k*. This approach results in a unique equilibrium solution, which provides the occupancy values for the conformational states.

For this study, we performed a new parametrization of the model using data from six structural pairs representing different variants of the Spike protein: ‘wild-type’ (PDB 7KDG and 7KDH; Gobeil et al. 2021a), D614G (PDB 7KDI and 7KDJ; Gobeil et al. 2021a), Beta (PDB 7WEV and 7VX1; Wang et al. 2021), Kappa (PDB 7VXI and 7VXE; Wang et al. 2021), Delta (PDB 7W94 and 7W92; Wang et al. 2022), and Omicron (PDB 7WK2 and 7WVN; Hong et al. 2022). The occupancy values associated with these structures were used to optimize the parameters *k* and *γ* for our system. The selected constants, *k *= 0.1 and *γ *= 0.1, yielded a Pearson’s correlation of 0.657 with the experimental occupancy values, showing good predictive accuracy.

Using this re-parameterized model, we derived a linear transformation to relate the calculated occupancy difference between open and closed states to the experimental values:


(2) $${\left( {{\mathrm{Open - Closed}}} \right)_{{\mathrm{Experimental}}}}{\mathrm{ = 1}}{\mathrm{.6237 \times }}{\left( {{\mathrm{Open - Closed}}} \right)_{{\mathrm{Calculated}}}}{\mathrm{ + 0}}{\mathrm{.0728}}$$

This transformation allows us to quantitatively compare the model’s predictions with observed experimental data with a robust mechanism for evaluating the conformational dynamics of the Spike protein. This method was employed to 180 homotrimeric structures of Spike, organized in pairs, as described above (see Section 2.1).

### 2.6 Longitudinal analysis of antibody escape and Vibrational Difference Score

Global longitudinal genomic data analysis of SARS-CoV-2 was performed using SPEAR v2 1 (Crown et al. 2022) against all consensus genome sequences deposited in GenBank (Benson et al. 2018) (accessed 17 August 2024) and marked as complete. A total of 3 048 694 samples were available for analysis with SPEAR. SPEAR integrates deep mutational scanning (DMS) data on antibody recognition and predicted Vibrational Difference Score (VDS) into a number of scores which can be tracked longitudinally.

From the GenBank dataset (*n* = 3 048 694), 2 994 442 met QC cutoffs and were analysed using SPEAR. Some samples do not have a valid collection date (e.g. Omicron samples occurring in 2020), or samples were specific to a particular year (which is not informative for longitudinal analysis). Samples were filtered to only include lineages where a collection date was present and was less than 100 days prior to lineage designation and had a sample date specific to a year and month at a minimum, resulting in a total of 2 841 216 samples being retained, spanning from December 2019 to August 2024. Samples were assigned to 3353 different Pangolin lineages, corresponding to 43 Nextstrain clades and 13 Variants of Concern (VOCs) (and a not VOC class).

From all 2 994 442 analysed samples, representative VCF files for 2941 lineages were created and analysed (requiring a minimum of five samples assigned to a lineage for analysis reduces the total number of lineages), using the SPEAR utilities-representative function. For a mutation to be included as representative of a lineage, it must occur within 50% of all samples assigned to a lineage. The lineage representative VCF files were then reanalysed using SPEAR in VCF mode in order to explore the functional effects of characteristic mutations of these lineages exhibit.

### 2.7 Determination of factors influencing trade-off

The trade-off analyses compare occupancy, antibody binding, and receptor binding calculations pairwise for particular mutations or sets thereof, such as variants. We utilized bootstrapping in order to account for differences in the amount of available data. For each pair, we built four datasets, containing data points from structures representing ‘wild-type’ and each variant for the two functional properties. From these datasets, we generated 500 bootstrap samples and calculated the difference between the variant and ‘wild-type’ for the mean value of each functional property.

## 3. Results and discussion

### 3.1 Antibody recognition

**Figure 1.**
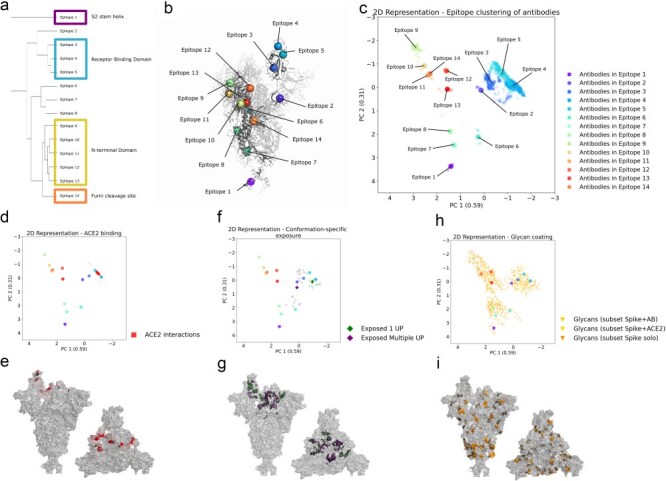
Schematic representation of epitope clustering and structure mapping. (a) Dendrogram representing the hierarchical clustering of SARS-CoV-2 Spike protein epitopes based on their average positions, (b) highlighted as coloured spheres in the open chain of the Spike structure, with each colour corresponding to a distinct epitope cluster. (c) Two-dimensional representation of antibody binding data using PCA, with respective variances indicated, showing the clustering of antibodies based on the distance of each average point of interaction to each epitope average position on the Spike protein. (d) The same technique for two-dimensional representation was employed to plot 209 ACE2-binding interactions (red spheres), showing the proximity to each epitope centre. (e) We can also see the mapping in the Spike surface of the sum of ACE2 vectors of interaction. (f) Another PCA plot shows residues with conformation-specific exposure, dependent on at least one RBD up (green diamond) or multiple RBD up (purple diamond); the average position of these residues is represented by larger markers for the same two groups. (g) Residues with conformation-specific exposure shown in the Spike surface. (h) PCA representation of glycan interactions for the subsets of structures of Spike in complex with antibodies (gold triangle), Spike in complex with ACE2 (yellow triangle), or unbound Spike (orange triangle). (i) Glycan coating for the unbound Spike subset mapped to the Spike protein surface.

Epitope characterization plays a key role in understanding immune recognition of the SARS-CoV-2 Spike protein and the mutations that disrupt antibody interactions. Accurately defining epitopes is crucial for determining the antibodies that need to be included to assess the impact of mutations on immune escape. Based on 2032 interaction vectors between Spike and antibodies (Section 2.3), we assigned each complex to one of the 14 different epitopes (Fig. 1a, b, and d) using their weighted representative point (Section 2.4).

The most common epitopes, 3, 4, and 5 (Supplementary Fig. S2A), describe interactions with the RBD, but with different orientations: Epitope 3 is characterized by strong interactions with positions 377 and 378 (Supplementary Fig. S2E). Epitope 4 is governed by residue 486 (Supplementary Fig. S2F), with interactions encompassing the region of the RBM. The strongest interacting residue in epitope 5 is 346, a residue that is considerably accessible even in closed conformations (Supplementary Fig. S2G).

Epitopes 9, 10, 11, 12, and 13 are localized within the N-terminal domain (NTD). This domain, located on the external side of the homotrimeric Spike structure, adopts a β-sandwich fold and forms a stable core. One of its sides faces the RBD of the same monomer, while the other side and the top of the domain are close to the RBD of the neighbouring monomer. Epitope 9 shows interactions in the external tip of the NTD, namely residue 147 (Supplementary Fig. S2K). Lower to the tip is a part of the NTD that constitutes the epitope 10, with governing residues 214 and 218 (Supplementary Fig. S2L). Epitope 11 characterizes a lateral portion of the NTD, closer to the neighbouring chain, mainly residues 173 and 176 (Supplementary Fig. S2M). The other lateral portion of the NTD forms a binding pocket along with the post-RBD area of the same Spike chain, creating epitope 12, with governing residues 85 and 237 (Supplementary Fig. S2N). Epitope 13 is located on the extreme lower part of NTD and is often characterized by buried peptides, mainly positions 277, 269, and 270 (Supplementary Fig. S2O).

Epitope 1 characterizes the interactions in the S2 stem helix, mainly residues 1148 and 1156 (Supplementary Fig. S2C). Epitope 2 is characterized by interactions in the external surface of Spike chains, after the RBD, with its main interacting residue being number 537 (Supplementary Fig. S2D). Epitopes 6 (Supplementary Fig. S2H), 7 (Supplementary Fig. S2I), 8 (Supplementary Fig. S2J), and 14 come mainly from structures of antibodies interacting with Spike peptides that are buried into the structure in the context of the entire homotrimeric protein. Another characteristic of epitope 14 is that it describes interactions with the furin cleavage site peptide (Supplementary Fig. S2P). For epitope 8, we also see interactions to the homotrimeric structure in the cases of glycan-mediated antibody binding (Supplementary Fig. S6C). The main interacting positions of these epitopes are described in Table 1.

**Table 1.** Main Spike residues for each epitope.

Epitope	Main Spike residues
1	1156, 1148, 1155, 1152, 1149, 1151, 1157
2	537
3	378
4	486, 489
5	346
6	1008, 1001, 765, 767, 1007, 1000, 772
7	904, 918, 898, 911, 917, 896, 897, 914, 912, 910
8	823, 815, 819, 822, 820
9	147, 248, 249, 246, 144, 146
10	214, 97, 69
11	173, 176, 175
12	237, 85, 83
13	277, 270, 269, 275, 271, 276, 274, 273
14	685

Residues with a mean interaction of −0.75 kcal/mol or lower, ordered from stronger to weaker mean binding affinity.

An important caveat is that many antibodies interact with more than one Spike chain simultaneously. For the purpose of assigning and defining epitopes, however, we consider only those with the Spike chain with which most significant interactions occur (based on Surfaces calculations). Of the 2032 antibody interaction vectors, when filtering out crystallization artefacts, there are 338 additional interactions with neighbouring Spike chains (Supplementary Fig. S3A). On average, the calculated absolute value of interaction pseudo-energy with these neighbouring chains is 7% of that with the primary interacting Spike chain. When classifying the secondary interactions according to the epitopes they target, we see the majority of the interactions with the neighbouring RBD, mainly epitope 3 (Supplementary Fig. S3B), but also with the neighbouring NTD (Supplementary Fig. S3C). A notable example of dual interaction is the antibody S2M11, which is classified as interacting with epitope 4 but also binds to the neighbouring epitope 3 with up to 69% of the binding affinity observed for the primary interacting chain.

Several epitope classifications exist (Piccoli et al. 2020, Sankhala et al. 2024), underscoring the value of epitope classification in SARS-CoV-2 research. However, the classification of Barnes et al. (2020a, 2020b) is the most widely adopted as a framework for studying antibody interactions. Although Barnes’ classification offers a concise overview of RBD epitopes, it (and others cited above) lacks practical tools for classifying novel complexes and excludes epitopes outside the RBD. Since the initial establishment of these classes in 2020, the volume of structural data available has grown significantly, allowing for a more robust and data-driven approach to epitope determination that addresses the limitations of existing classifications, including that of Barnes.

**Figure 2.**
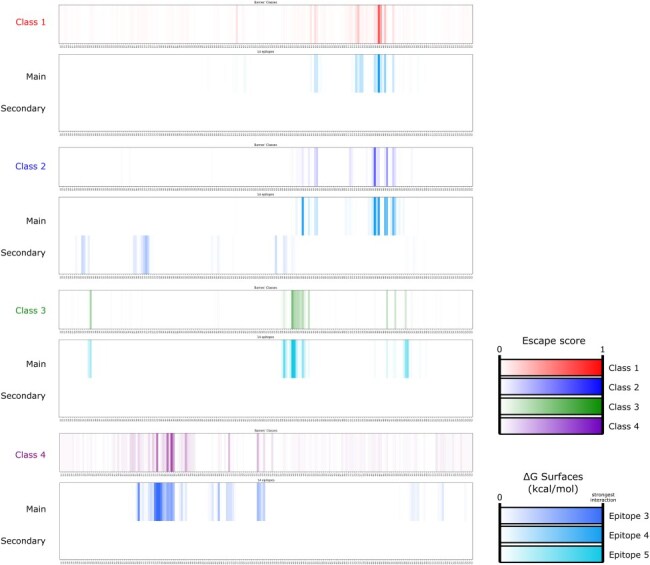
Comparative analysis of epitope classifications. Comparative analysis of epitope classifications between Barnes et al. (2020a, 2020b) and our data-driven epitope determination approach. Each row represents an epitope class from Barnes’ classification (classes 1–4), represented by the site escape scores for each RBD residue, and the respective pseudo-energies calculated via Surfaces, showing interaction strength across different epitopes (epitopes 3, 4, and 5). Class 1 is represented by escape data from antibodies S2E12, COV2-2196/AZD8895, REGN10933/casirivimab, and C102 and is equivalent to interaction data characterizing main interactions to epitope 4 from structures 7K45, 7K4N, 7R6X, 7L7D, 7L7E, 6XDG, and 7K8M. Class 2 is represented by escape data from antibodies LY-CoV555/bamlanivimab, S2M11, C002, C144, and C121 and is equivalent to interaction data characterizing main interactions to epitope 4 and secondary interactions to epitope 3 from structures 7L3N, 8DLW, 7K43, 7SO9, 7LXY, 7K8S, 7K8T, 7K90, 7K8X, and 7K8Y. Class 3 is represented by escape data from antibodies REGN10987/imdevimab, COV2-2130/AZD1061, C135, LY-CoV1404/bebtelovimab, and C110 and is equivalent to interaction data characterizing main interactions to epitope 5 from structures 8J26, 8J1Q, 7ZJL, 8SUO, 7L7E, 7K8Z, 7MMO, and 7K8V. Class 4 is represented by escape data from antibodies CR3022, COVA1-16, and S304 and is equivalent to interaction data characterizing main interactions to epitope 3 from structures 7A5R, 6W41, 7LM8, 7S5Q, 7JW0, 7X1M, and 7R6X.

Given the widespread use of this classification, we assessed the equivalence of our own categorized epitopes to Barnes’ four classes by analysing epitopes targeted by a set of well-documented antibodies with experimentally determined structures in complex with the Spike protein. Using Surfaces to calculate per-residue pseudo-energies, we found a strong correlation between residues crucial for each interaction and the total escape per site calculated for the same antibodies based on DMS results (Greaney et al. 2022, Cao et al. 2023) (Table 2). By examining these metrics across each class and epitope, we observed alignment between Barnes’ classes 1 and 2 with our epitope 4, class 3 with epitope 5, and class 4 with epitope 3 (Fig. 2). Notably, while classes 1 and 2 show substantial overlap in escape data characterizing critical residues for antibody recognition, their distinction arises from a secondary interaction with an adjacent Spike chain—a feature not apparent in escape assays due to the experimental use of expressed RBD rather than the full homotrimeric Spike complex, and a distinction our interaction evaluations are able to capture (Fig. 2).

**Table 2.** Epitope classification equivalence.

Antibody	Barnes’ class	Epitope	PDB ID	Pearson’s correlation
S2E12	1	4	7K45, 7K4N, 7R6X	−0.86
REGN10933/casirivimab	1	4	6XDG	−0.81
COV2-2196/AZD8895	1	4	7L7D, 7L7E	−0.80
C102	1	4	7K8M	−0.62
LY-CoV555/bamlanivimab	2	4	7L3N	−0.88
S2M11	2	4	8DLW, 7K43, 7SO9, 7LXY	−0.76
C002	2	4	7K8S, 7K8T	−0.75
C144	2	4	7K90	−0.63
C121	2	4	7K8X, 7K8Y	−0.53
REGN10987/imdevimab	3	5	8J26, 8J1Q, 7ZJL	−0.75
COV2-2130/AZD1061	3	5	8SUO, 7L7E	−0.75
C135	3	5	7K8Z	−0.73
LY-CoV1404/bebtelovimab	3	5	7MMO	−0.69
C110	3	5	7K8V	−0.63
CR3022	4	3	7A5R, 6W41	−0.66
COVA1-16	4	3	7LM8, 7S5Q	−0.58
S304	4	3	7JW0, 7X1M, 7R6X	−0.52

Beyond establishing the equivalence with Barnes’ classes, our evaluation demonstrates that per-residue contributions as calculated with Surfaces can predict DMS-based immune escape calculations. Specifically, experimental immune escape scores (Greaney et al. 2022, Cao et al. 2023) (ranging from 0 to 1) align with per-residue interactions (measured as pseudo-energy, where more negative values indicate stronger interactions). Table 2 shows the correlation between the average per-residue immune escape score and Surfaces’ interaction energy predictions for several instances of different antibodies. The Pearson’s *R* obtained ranges from −0.52 to −0.86. It is noteworthy that the average correlations are markedly lower for antibodies in class 3 (Barnes class 4). This alignment emphasizes the predictive accuracy of Surfaces in identifying immune escape potential across different Spike residues and Spike-targeting antibodies.

This comparative evaluation could be extended to all antibodies with available structures in complex with the Spike protein and included in DMS studies, provided that the nomenclature of these antibodies is standardized. As a preliminary assessment, the results we present for the 18 antibodies demonstrate promising predictive accuracy.

**Figure 3.**
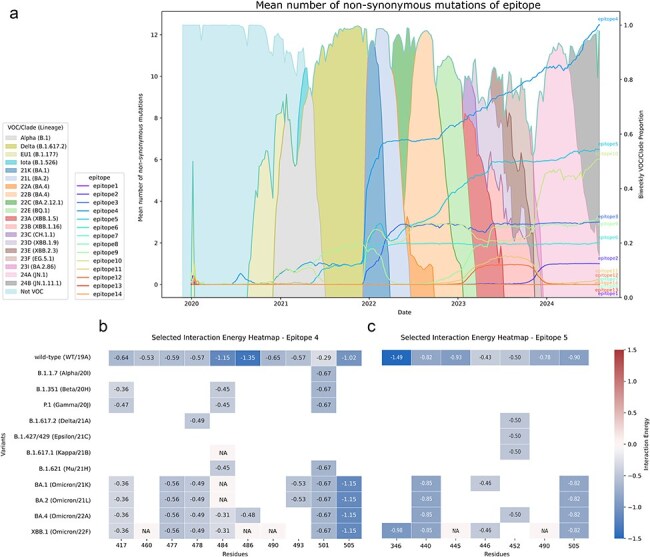
Evolution of non-synonymous mutations and interaction energies in Spike protein epitopes. (a) Timeline of mean missense mutations in 14 Spike protein epitopes across SARS-CoV-2 variants from 2020 to 2024, plotted over the bi-weekly VOC/clade proportion. The VOCs and clades making up more than 10% of samples within each bi-weekly interval are shown. (b) Per-residue interaction energies for the mutated residues across various SARS-CoV-2 variants for epitope 4 and (c) epitope 5, with energy values (kcal/mol) calculated using Surfaces. Darker shades of blue indicate stronger favourable interactions. NA: data not available.

The longitudinal plot shown in Fig. 3a shows the number of mutations that the 14 characterized epitopes accumulated in the timeline of the pandemic. Starting in the Alpha variant, which did not exhibit significant antibody escape (Collier et al. 2021), we see mutations starting to accumulate on epitope 4, with the substitution N501Y, that presented an increase in overall binding affinity in structural evaluations (Fig. 3b). The emergence of Delta coincides with an increase in immune escape, driven by a number of mutations to Spike (Mlcochova et al. 2021) including L452R (Li et al. 2020), part of epitope 5. The most significant jump in possible antibody escape seen in Fig. 3a corresponds to the early Omicron Nextstrain clades 21 K and 21 L.

The Omicron VOC is the most highly mutated SARS-CoV-2 VOC to date (Fan et al. 2022), with 38 Spike protein mutations. The significantly altered Spike protein results in a large increase in immune escape even when compared to Delta (Planas et al. 2022, Willett et al. 2022). This jump can be observed across many epitopes but is particularly apparent in epitopes 4, 5, 10, and 6. Following this initial jump, a number of Omicron Nextstrain subclades and Pangolin lineages have emerged, with small numbers of mutations aggregating when compared to the basal 21 K/21 L clades.

Among the epitopes, epitope 4 has undergone the most mutations over time, followed by epitope 5. This pattern is evident as we move into the recent JN.1 and JN.1.11.1 lineages (clades 24A and 24B), which have dominated the sampled population since the start of 2024, comprising 91.28% of analysed samples from 2024 on. JN.1, with a single mutation at L455S compared to its ancestor BA.2.86, shows a notable increase in antibody evasion, particularly against Barnes’ class 1 and 2 antibodies (Paciello et al. 2024), equivalent to epitope 4. Using the complexes with antibodies targeting epitopes 4 and 5 in variants until the recombinant XBB.1, we can pinpoint the mutations that impacted the binding affinity for those complexes. In epitope 4, substitutions in positions 417, 484, and 486 create the largest decrease in binding affinity to antibodies. For epitope 5, the R346T mutation, evaluated for its interactions in the context of the XBB.1 variant, considerably decreases antibody recognition, in accordance to experimental evaluations (Groenheit et al. 2023, Chakraborty et al. 2023a).

While mutations in epitope 5 plateaued after the emergence of XBB sublineages, epitope 10 has shown a notable rise in mutations in 2024 (Fig. 3a), with substitutions such as N211I and H245N, and deletions at positions 27 and 212, over backgrounds that already carried V213G and del69. These changes underscore the adaptive pressures on regions outside the RBD, specifically in the NTD, where epitope 10 resides. However, due to the limited availability of complexes involving antibodies targeting epitope 10, it is challenging to assess the exact effects of these mutations on immune recognition based on structural evaluations.

This shift suggests that mutations in non-RBD epitopes like epitope 10 may also contribute to immune evasion, emphasizing the importance of studying and classifying epitopes beyond the RBD. Understanding mutation patterns in epitopes that experience fewer substitutions is equally valuable. Epitopes with limited mutations may serve as stable targets for antibody binding, representing potential avenues for therapeutic and vaccine design.

In our structural analysis, we differentiated antibody complexes by variant to identify potential patterns of immune recognition over the course of the pandemic in the overall interactions. A key limitation of this approach is that it does not account for the origin of the antibodies or the time when they first emerged, meaning the specific variant against which these antibodies initially arose is not considered. This limitation may disproportionately affect the evaluation of the earlier VOC discussed here.

In the case of the Alpha variant, we observed increased antibody recognition for epitopes 4 and 5 (Supplementary Fig. S4B), although epitope 5 is represented by only one vector, limiting its significance (Supplementary Fig. S4A). The increased antibody binding affinity to epitope 4 is associated with the N501Y mutation. For the Beta variant, while the N501Y mutation enhances binding affinity at epitope 4, the overall interaction at this epitope is affected by mutations at positions 417 and 484 (Fig. 8a), leading to decreased immune recognition, as previously documented (Francino-Urdaniz et al. 2021, Zhang et al. 2021, Greaney et al. 2022). Additionally, we noted increased binding affinity for epitope 9, which involves interactions with mutated positions 18 and 246, and epitope 3 (Supplementary Fig. S4B). Regarding the Gamma variant, we observed increased binding affinity for epitopes 3, 4, and 9 (Supplementary Fig. S4B), although these findings are based on very few vectors and therefore not representative (Supplementary Fig. S4A). For epitope 9, e.g. we have one vector interacting with Fab 4A8 antibody, showing lower binding affinity than ‘wild-type’ and stronger binding affinity than BA.2 for the same antibody, while for Fab 4-8 antibody we see Gamma interacting with stronger affinity than ‘wild-type’. EY6A, the only epitope 3 antibody we see interacting with Gamma, and COVOX-222, the only antibody targeting epitope 4 interacting with Gamma, show similar interactions when in complex with ‘wild-type’, Alpha and Beta.

We also have a small subset of structures for the Mu variant, making the evaluation of the decreased binding affinity for epitope 3 less reliable (Supplementary Fig. S4A). Also, the Mu interaction results are based only on interactions with antibody VACW-209, and the binding affinity is similar to that of the same antibody with ‘wild-type’ Spike and BA.1 variant. The results for the Delta variant show stronger binding affinity in epitopes 3 and 4 (Supplementary Fig. S4B). Considering particular antibodies enriched in the dataset, we see stronger interactions of Delta with S2L20 and VACW-209 when compared to ‘wild-type’ and other variants.

For the Kappa variant we see, at first, a significant decrease in binding affinity for epitope 3 (Supplementary Fig. S4B), which is not coherent with the positions of its mutations, L452R and E484Q, located at epitope 4. Inspecting the data closely, we see that all Kappa interactions on epitope 3 are of antibody S309, the antibody with the highest representation in our dataset across different variants. Comparing all the vectors of interaction for S309, we see that one Kappa structure (PDB 7SOB), from which three out of the four vectors of interaction in epitope 3 come, is not in contact with positions 354–361 due to a slightly different geometry of interaction. We also see this for Omicron structures (PBD 7TM0, 7YQY), but this effect gets diluted for variants with larger subsets of structures. The effect we see, therefore, is not related to Kappa mutations. For epitope 12, all Kappa interactions are with antibody S2L20, also present in our dataset in complex with other variants. When comparing only S2L20 interactions, we see Kappa, Delta, and BA.4/BA.5—considered together due to identical mutational profiles for the Spike protein—sharing similar interactions, while Epsilon and BA.1 show decreased binding affinities.

For the BA.1 variant as a whole, there was a significant decrease in binding affinity observed for epitope 3 in general (Supplementary Fig. S4B), located near the mutated position 375. Interestingly, we see stronger binding affinity for BA.1 with antibody EY6A when compared to ‘wild-type’ and other variants. The BA.4/BA.5 variant showed a significant decrease in overall strength of interactions for epitope 4, which is primarily due to the crucial mutation at position 486 (Qu et al. 2023), along with substitutions T478K, Q498R, and S477N. Lastly, the XBB.1 variant exhibited a significant decrease in binding affinity for epitope 3 (Supplementary Fig. S4B), although this observation is based on only one vector of interaction of a complex with S309.

In summary, we present a comprehensive classification of 14 epitopes covering the majority of the Spike protein. This classification, based on 942 Spike–antibody complexes, provides a robust framework for the automated analysis of new complexes with a clear and replicable methodology. The evaluation of per-residue antibody recognition, performed with Surfaces, showed high correlation with escape scores calculated from DMS results. Although our per-epitope per-variant evaluation is limited by the varying compositions of the subsets, it suggests potential patterns of immune recognition and escape, particularly highlighting epitope 3 as frequently associated with significant variations in binding affinity across different variants. SPEAR was used to evaluate longitudinal trends in variants, allowing exploration of the mutations arising in variants not represented in the structural dataset, which highlighted epitopes 4 and 10 as being of interest.

### 3.2 Receptor-binding affinity

The primary functional role of the Spike protein in SARS-CoV-2 cell entry involves its interaction with the receptor ACE2. Each vector of Spike–ACE2 interaction was classified into the corresponding epitope. Of the total of 209, 206 vectors are assigned to epitope 4 (Fig. 1d and e).

**Figure 4.**
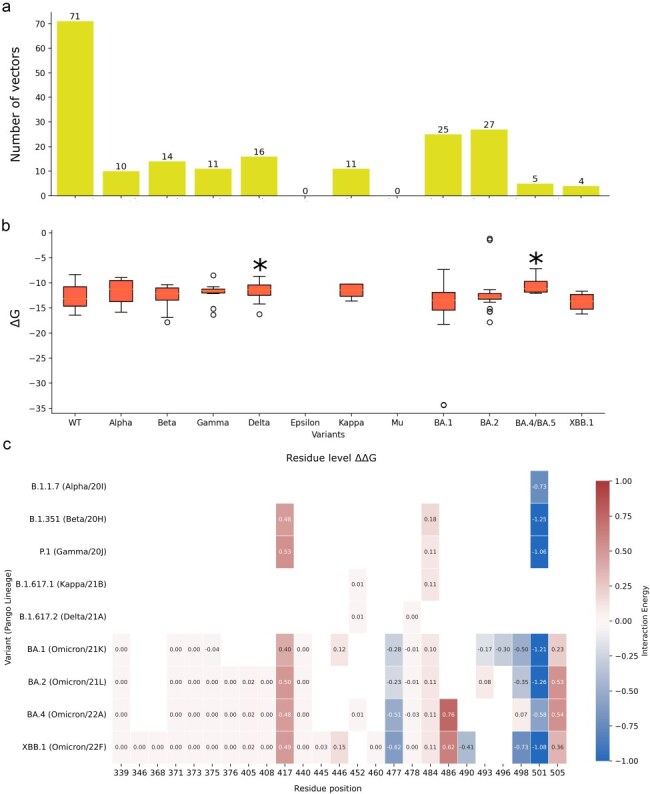
ACE2-binding calculations. (a) Composition of the dataset, showing the number of interaction vectors per variant. (b) Calculated total ACE2 interactions (kcal/mol) for structures representing each variant; *P < .05. (c) Per-residue differences in average interaction between each evaluated variant and the ‘wild-type’ ACE2 interaction for the mutated positions.

Spike–ACE2 complexes were evaluated according to the variant they represent based on annotations from the structural dataset. Alpha, Beta, and Gamma are consistently associated with higher ACE2 binding in experimental results (Barton et al. 2021, Cameroni et al. 2022, Han et al. 2022), particularly due to the Y501-mediated increased binding (Starr et al. 2020, Laffeber et al. 2021, Tian et al. 2021, Geng et al. 2022, Moulana et al. 2022, Sergeeva et al. 2023). In our results, the N501Y mutation is shown to increase binding affinity, but at the same time we see lower binding for positions 496 and 498, that compensates the 501 mutation effects (Fig. 4c). The mutations on position 417, first introduced in Beta and Gamma, also appear to decrease binding according to our interaction evaluations (Fig. 4c), an observation also seen through other computational (Jawad et al. 2021, Sergeeva et al. 2023) and experimental evaluations (Starr et al. 2020, Laffeber et al. 2021). All variants carrying mutations on position 484 show minor effects in binding affinity on this position, also in agreement with the literature (Starr et al. 2020, Jawad et al. 2021, Laffeber et al. 2021, Sergeeva et al. 2023).

The only two variants that we were able to map as having significantly different total values of interaction compared to ‘wild-type’ were Delta and BA.4/BA.5, both with decreased binding affinity to ACE2 (Fig. 4b). Experimental data on the binding of Delta to ACE2 are conflicting but mostly show that they did not present much difference when compared to ‘wild-type’ (Cameroni et al. 2022, Han et al. 2022, Schubert et al. 2022), which is consistent with the minor effects reported for each of its mutations individually (Starr et al. 2020, Sergeeva et al. 2023). Delta, characterized by the mutations L452R and T478K in the RBD, shows in the per-residue evaluation a decrease in binding affinity, particularly in position 493, which is in close proximity to position 452 in the folded RBM. On the other hand, Kappa, which also brings the L452R substitution, as well as E484Q, shows milder effects on position 493 and slightly stronger effects in position 490, also in the neighbouring region of 452. Still, it shows the smallest differences in the per-residue binding decomposition when compared to ‘wild-type’ among all the variants analysed. For both variants, we see increased binding in positions 486 and 487, which are close to the mutated K478 in Delta and the mutated Q484 in Kappa.

Mutations on position 486, present in variants BA.4/BA.5 and XBB.1, show decreased binding affinity to ACE2 (Fig. 4c), in agreement with experimental evidence (Starr et al. 2020, Cao et al. 2022, Sergeeva et al. 2023, Zhang et al. 2023). The same is true for the Y505H substitution (Fig. 4c), present in Omicron subclades (Geng et al. 2022, Kimura et al. 2022). The S477N substitution, also part of the Omicron subclades, is associated with increased binding affinity, both in the per-residue decomposition of our analyses (Fig. 4c) and in different experimental evaluations (Geng et al. 2022, Sergeeva et al. 2023). The Q498 mutation, associated with unfavourable interactions in the context of the N501Y mutation for variants Alpha, Beta, and Gamma, once mutated to R498 in the Omicron subclades, shows a significant contribution to increased binding complementarity for BA.1, BA.2, and XBB.1 (Fig. 4c) (Moulana et al. 2022, Kim et al. 2023)^.^

In summary, we examined the Spike–ACE2 interaction across 209 interacting interfaces from 146 structures. From those evaluations, we can characterize epitope 4 as the shared binding site between ACE2 and antibodies. Our variant-specific analysis highlighted the complex, context-specific and sometimes compensatory effects of key mutations like N501Y, K417N/T, S477N, Q498R, F486V/S, and Y505H.

### 3.3 Conformational dynamics

The conformational dynamics of the Spike protein impact several of its functionalities. To evaluate the dynamical modulation of antibody recognition and receptor binding, we first classified the 14 epitopes based on their conformation-dependent exposure. We calculated interaction vectors between Spike chains within the trimer, in which the interacting chains could be in different conformations. Analysing these vectors, we observe that some residues that belong to particular epitopes only become exposed when one RBD is in the open conformation and likewise, there are residues that only become exposed when two chains of Spike in the open-state interact. To allow for visual comparison, we map these two cases onto the same reference structure of Spike used for all representations in Fig. 1 (with one open RBD) (Fig. 1g). Calculating the distances between the position of the residues that are exposed when at least one RBD is open (in green in 1 G), to the position of epitope centres (Fig. 1b), we can see that the exposure of epitope 4 is contingent upon at least one RBD being in the open conformation (Fig. 1f), consistent with the observation that epitope 4 includes the RBM. Figure 1g also shows the residues that only became exposed when multiple RBDs are open (in purple). As can be seen in Fig. 1g, some of these in a darker shade of purple (see also the available PyMOL session, link provided in the Data availability section) are actually occluded and would only be exposed if they were mapped onto the homotrimeric Spike structure with two RBD in the open conformation. These residues are mostly part of epitope 3 (Fig. 1f). The conformation-dependent exposure of epitope 3 can be visualized in Supplementary Fig. S2E, in which the alignment of the interacting nanobody 17 F6 (PDB 7FBJ) to the reference one-RBD-up Spike structure creates overlaps, that would prevent this binding site to exist due to steric clashes.

**Figure 5.**
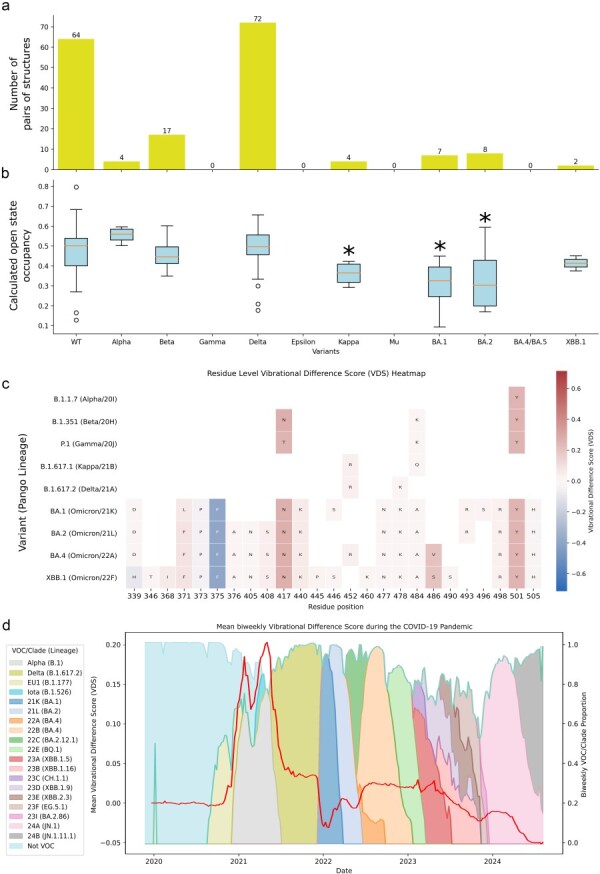
Occupancy calculations. (a) Dataset composition with the number of pairs of structures per variant. (b) Calculated open-state occupancy for the pairs of structures representing each variant, *P < .05. (c) VDS values for the mutations that constitute each variant (data from Teruel et al. 2021b), and (d) the mean bi-weekly VDS evolution during the COVID-19 pandemic, plotted over the bi-weekly VOC/clade proportion. The VOCs and clades making up more than 10% of samples within each bi-weekly interval are shown.

We selected pairs of structures representing the closed conformation and the one-RBD-open conformation, to allow us to calculate the occupancy of each of these two states for different variants (Fig. 5a). This evaluation is important to estimate the exposure of these epitopes in each variant represented in our dataset of structures. We calculated the open-state occupancy for each pair of structures (Fig. 5b) employing the same methodology previously used to evaluate modelled single mutations (Teruel et al. 2021a), with an updated parameterization of the Markov chain (see Section 2). ‘Wild-type’ and Delta are the two groups with the most pairs of structures: 64 and 72, respectively. The high number of structures, carrying many structural variations, brings along a high dispersion of results. It is important to notice that many publications focused on experimentally investigating conformational states for different Spike variants describe substates and subpopulations (Gobeil et al. 2021a, 2021b, 2022) that we did not consider in the evaluation; for the purpose of comparing variants, the conformational states to be compared need to be equivalent. For that reason, these substates are based on a more simplistic evaluation of closed and open, possibly contributing as well to data dispersion. From the previously modelled single mutations, we also present the VDS, a proxy for occupancy based on gain or loss of flexibility in different conformational states, for the mutations that constitute each of the variants in an attempt to interpret the reasons for occupancy shifts evaluating the effects of single substitutions (Fig. 5c).

Based on the distribution of points representing pairs of ‘wild-type’ Spike structures, we do not observe a significant difference in the open-state occupancy of the Alpha variant. The average calculated open-state occupancy, however, is higher than ‘wild-type’. This result aligns with previous calculations for modelled Alpha structures (Teruel et al. 2021a), as well as with the sharp increase in sample level VDS, derived through SPEAR, corresponding to the emergence of Alpha VOC as seen in Fig. 5, due to the N501Y mutation increasing the flexibility of the closed Spike and the rigidity of the open state (Teruel et al. 2021b). This observation is consistent with experimental measurements (Gobeil et al. 2021a).

Even with the very high dispersion of the ‘wild-type’ data, used to determine significance, a significant reduction in the open-state occupancy of Kappa, BA.1 and BA.2 variants is seen. The Omicron subclades contain the important S375F substitution (Kimura et al. 2022), which presents a strongly negative VDS (−0.39 VDS, Fig. 5c), meaning it stabilizes the closed conformation according to our previous evaluations (Teruel et al. 2021b). This observation is reflected in the longitudinal analysis of VDS (Fig. 5d), in which a drop can also be seen with the onset of the Clade 21 K (BA.1) Omicron wave, driven by the acquisition of the S375F mutation (Fig. 5c). Experimental results also show a stabilized closed conformation in Omicron subclades (Cao et al. 2022, Gobeil et al. 2022, Hong et al. 2022) and pinpoint the role of S375F in this functional shift (Zhao et al. 2022)^.^

We do not see in Fig. 5b the same pattern of decreased open-state occupancy for XBB.1, resulted from recombination between two lineages of BA.2 (Chakraborty et al. 2023b)—we see, instead, fluctuations in the calculated open-state occupancies of different variants. One extra substitution that characterizes XBB.1 and was associated with increased open occupancy as a modelled single mutant is F486S (Fig. 5c) (Teruel et al. 2021b).

Using longitudinal analysis of mutations affecting VDS, the trends observed in structures demonstrated in Fig. 5b can be extended to the latest circulating variants. Here, it’s relevant to highlight substitutions L455S, L455F, and F456L, part of the JN.1-derived subvariants SLip and FLiRT, that present negative VDS (Teruel et al. 2021a), possibly contributing to the trend of stability of the closed conformation (Pei et al. 2024), as well as to immune escape for antibodies targeting conformation-dependent epitopes.

To summarize, we mapped epitopes 3 and 4 as conformation-specific epitopes by comparing the positions of the 14 predetermined epitopes with that of residues that exhibit conformation-dependent exposure. Our per-variant occupancy calculations suggest an initial increase in open-state occupancy, primarily driven by the dynamical effects of the N501Y mutation (Fig. 5c), followed by a decrease in open-state occupancy in the Omicron subclades, largely due to the effects of the S375F mutation (Fig. 5c), and to substitutions in positions 455 and 456 for SLip and FLiRT.

### 3.4 Glycan coating

Filtering the structures for the presence of glycans shows that the vast majority are glycosylated (Table 3). This extensive glycan coating covers various parts of the protein (Fig. 1c and g). Notably, we do not observe significant glycan coverage around the RBM, likely due to natural selection favouring receptor binding—an observation in accordance with previous studies (Grant et al. 2020). To ensure that protein binding near the RBM does not bias this observation, we defined subsets of structures based on the presence of other macromolecules and specifically evaluated the glycan interactions of the unbound Spike (Table 3). This analysis confirms that the lack of glycosylation in the RBM region is an inherent feature of the structure and not influenced by external binding partners (Fig. 1i).

**Table 3.** Composition of the dataset of structures regarding the presence and interactions of glycans.

Subset	Number of structures	Number of structures containing glycans (%)	Number of structures with non-null interactions between glycans and Spike (%)	Number of structures with glycans co-interacting with more than one chain of Spike (%)	Number of structures with non-null interactions between glycans and the interacting protein (%)	Number of structures with glycans co-interacting with Spike and its interacting protein (%)
Spike + antibody	942	723 (76.8%)	721 (76.5%)	101 (10.7%)	201 (21.3%)	184 (19.5%)
Spike + ACE2	146	118 (78.6%)	111 (76.0%)	39 (26.7%)	107 (73.3%)	16 (11.0%)
Unbound Spike	338	269 (80.8%)	269 (80.8%)	261 (77.2%)	–	–

Similar glycan coating patterns appear in the other two subsets—structures of Spike in complex with antibodies and with the receptor ACE2 (Supplementary Fig. S5A and B). In the antibody complexes, glycans coat key immune recognition epitopes (Supplementary Fig. S5C and D), raising the possibility that glycans contribute to immune escape. In glycosylated structures bound to ACE2, glycans interact with residues near the peripheral RBM region (Supplementary Fig. S5E and F), though the glycans attach to residues in neighbouring Spike chains rather than within the RBM itself, co-interacting with the closest RBD (Supplementary Fig. S5G). We also observe glycans co-interacting with Spike and antibody chains (Supplementary Fig. S5G). Their presence may not be obligatory, as some antibody complexes targeting the same epitope exhibit high surface complementarity without glycans (Supplementary Fig. S5I). Examples like these prompted us to search the entire dataset for co-interacting glycans to further investigate their role in immune recognition (Table 3).

**Figure 6.**
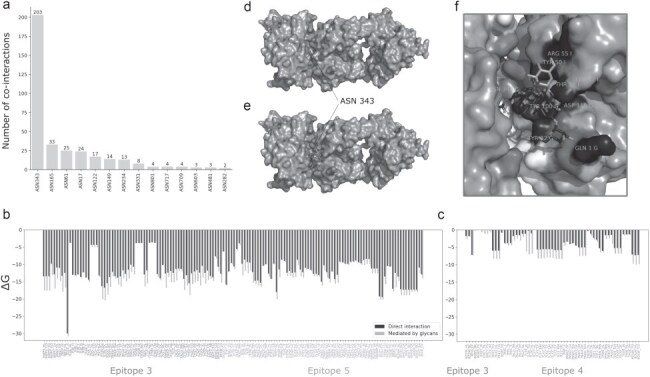
Characterization of glycan-mediated antibody binding. (a) Number of co-interactions per glycan-linked residue. (b) Values of binding affinity (∆*G*, kcal/mol) comparing direct interaction (blue) to interaction mediated by ASN343-linked glycans (orange) for co-interactions in which the glycan-mediated binding of antibody chains to its main interacting Spike chain, or (c) a neighbouring Spike chain. (d) Direct interaction between Spike RBD and the S309 antibody (PDB 7BEP), and (e) the same interaction mediated by ASN343-linked glycan, showing an increased binding site. (f) Close-up of the delta between results, showing a gain of interactions for most antibody residues, namely GLN1, TYR32, TYR100, and ASP115 from the S309 heavy chain and LEU471, TYR501, and ARG551 from the S309 light chain, and a loss of direct interaction of Spike’s ASN343.

Investigating the effects of the most common co-interacting glycan, we identified 203 interaction vectors influenced by the glycan linked to asparagine 343 (ASN343) (Fig. 6a). In the main interacting Spike chain, this glycan increases the binding surface area by introducing new interacting residues, resulting in an average 12.65% increase in binding affinity, with the largest gain reaching 4.56 kcal/mol (Fig. 6d–f).

Considering the specific epitopes for each interaction, ASN343 glycosylation mediates interactions in epitopes 3 and 5. The evaluation of glycan mediation per epitope reveals an average interaction gain of 2.00 kcal/mol and 1.07 kcal/mol, as predicted by Surfaces, representing a 16.50% and 9.21% increase in affinity, respectively (Fig. 6b). Beyond enhancing interactions with the main interacting Spike chain, the ASN343-linked glycan significantly increases interactions with neighbouring Spike chains, especially for antibodies targeting epitope 4. This effect is pronounced in structures like PDB 7L56, where glycan mediation greatly multiplies interactions (Fig. 6c). Including these interactions, the largest binding affinity gain reaches 6.77 kcal/mol.

We also observed interactions being mediated by glycans linked to other asparagine residues (Supplementary Fig. S6A). Noteworthy examples include strengthened interactions between neighbouring chains, mediated by glycans linked to ASN165 and ASN122 (Supplementary Fig. S6B), glycan-dependent interactions of antibody 2 G12 targeting epitope 8 via glycans linked to ASN801, ASN717, and ANS709 (Supplementary Fig. S6C) (Acharya et al. 2020, Harvey et al. 2021), and enhanced interactions in antibodies targeting epitope 13 due to glycans linked to ASN61, ASN282, and ANS603 (Supplementary Fig. S6D).

All co-interacting glycans linked to Spike residues mediate antibody interactions. Some structures reveal glycans co-interacting with both Spike and ACE2, all linked to ACE2 residues (Supplementary Fig. S7A and C) and playing important roles in ACE2 recognition (Allen et al. 2021, Gong et al. 2021, Nguyen et al. 2021). We also observed a few antibody complexes where glycans link to residues within the antibody chains, though these cases are rare (Supplementary Fig. S7B and D).

Beyond mediating antibody interactions, we investigated whether glycans can mask specific epitopes. Some residues, such as ASN1074, ASN1098, ASN1134, and ANS1158 in epitope 1, are never glycosylated in the context of antibody binding to the same epitope—when evaluating each structure, the presence of these glycans and of antibodies bound to epitope 1 appears to be mutually exclusive, suggesting that the recognition of epitope 1 is masked by the glycan shield. Glycans only link to these residues in structures without antibodies in complex or where the antibodies target different epitopes.

**Figure 7.**
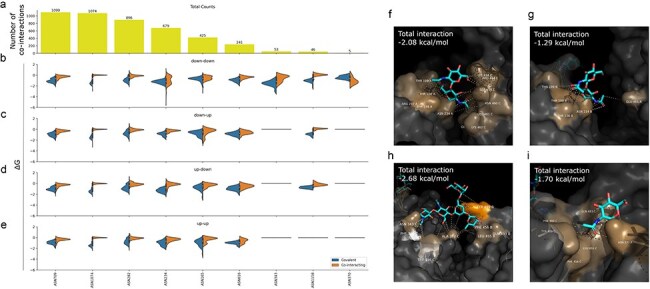
Characterization of glycan-mediated conformational stability. (a) Number of co-interacting glycans based on the Spike residue they are covalently linked to. (b) Distributions of binding affinity (∆*G*, kcal/mol) of glycans bound to a Spike chain in the down conformation and co-interacting with a Spike chain in the down conformation, (c) bound to a Spike chain in the down conformation and co-interacting with a Spike chain in the up conformation, (d) bound to a Spike chain in the up conformation and co-interacting with a Spike chain in the down conformation, and (e) bound to a Spike chain in the up conformation and co-interacting with a Spike chain in the up conformation, with the distribution of total affinity for the chain they are covalently bound to (blue) and the co-interacting chain (orange). For glycans linked to residue ASN234, the affinity to the co-interacting chain is much higher for chains in the down conformation (f) than in the up conformation (g), as exemplified by two different pairs of Spike chains from the same structure (PDB 6XM3). We can also see examples of glycans (h) linked to ASN343 (PDB 8EPN), as well as (i) to ASN370 (PDB 7FCE), stabilizing the interaction between a pair of closed Spike chains.

While selecting structures in our dataset in which there are glycans co-interacting with more than one protein unit, we identified many structures where glycans co-interact with more than one Spike protein chain. To assess their role in mediating conformational dynamics, we examined how frequently these interactions occur, along with the interaction values between the linked chain and the co-interacting chain, across different Spike conformational states (Fig. 7a–d). Glycans linked to ANS709 and ASN1074 often co-interact with neighbouring Spike chains, but the distribution of binding affinities remains unchanged across conformational states, as both positions are distant from the RBD. However, for ASN234-linked glycans, we observe conformation-specific results, with significantly stronger affinity for the co-interacting chain when both chains are in the down conformation (Fig. 7b, f, and g). Glycans linked to ASN343 and ASN370 also show conformation-specific interactions, occurring only when both chains are in the down conformation (Fig. 7b, h, and i). ASN234-, ASN343-, and ASN370-linked glycans had previously been described for their role modulating the opening of the RBD (Sztain et al. 2021, Harbison et al. 2022, Pang et al. 2022, Zhang et al. 2022); the same is true for ASN165-linked glycans, but our interaction results did not show conformation-specific changes in binding affinity. Based on the same evaluation of enriched results, the ASN1158 position can also be highlighted (Fig. 7e), but the absence of co-interactions between two Spike chains in the up conformation likely stems from the low resolution of the C-terminal in many homotrimeric structures.

**Figure 8.**
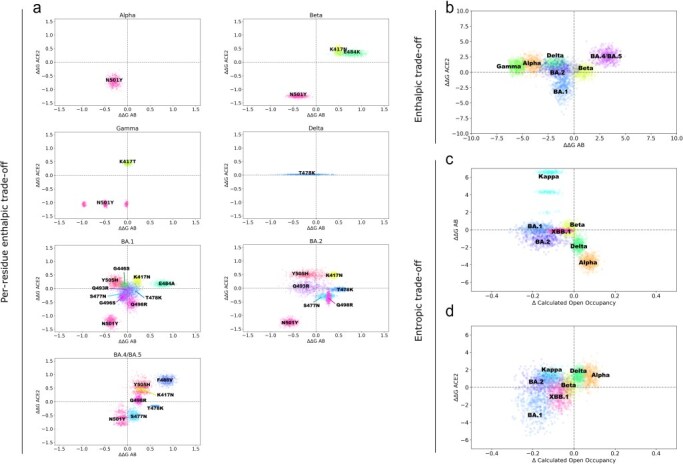
Comparative metrics of ACE2-binding affinity (∆∆*G* ACE2, kcal/mol), antibody recognition (∆∆*G* AB, kcal/mol), and conformational dynamics for Spike variants (∆ Calculated Open Occupancy, %). (a) Results of binding affinity with ACE2 and with antibodies for each mutated residue in the RBM of structures representing variants Alpha, Beta, Gamma, Delta, BA.1, BA.2, and BA.4/BA.5, and (b) full interaction results for the same structures. (c) Results of open occupancy calculations based on structures of variants Alpha, Beta, Delta, Kappa, BA.1, BA.2, and XBB.1 against the full interactions of the same variants with the receptor ACE2, or (d) with antibodies targeting conformation-specific epitopes.

Due to the inherent flexibility of glycans, their resolution is often limited in structural data. Since we did not manipulate the structures or reconstruct the entire glycan chains, this may have influenced our evaluations. By focusing only on the portions of glycans explicitly resolved in the structures, our analysis is inherently biased by the resolution of the data. However, the glycosylated positions we highlight with conformation-specific glycan interactions are typically evaluated within the same structure, comparing chains in different conformational states (Fig. 7f and g), and are therefore subject to consistent resolution constraints. Furthermore, the most flexible regions of the glycans are less constrained due to the absence of strong interactions, meaning these regions would not significantly contribute to our interaction evaluations.

Given the functional relevance of glycans linked to specific residues, we assessed their conservation to identify potential mutations that could disrupt glycosylation and affect function indirectly. All positions selected for their potential impact on protein dynamics and antibody recognition are highly conserved according to CoV-Spectrum data (Chen et al. 2022) (Supplementary Table S1), aligning with other computational studies that suggest Spike protein mutations typically occur in regions where antibody access is not obstructed by glycans (Ren von Bu¨low et al. 2023). Although our occupancy calculations did not account for glycan-mediated interactions between Spike chains, this exclusion does not affect our conclusions, as our analysis focused on vibrational entropy differences relative to ‘wild-type’ structures, which similarly excluded glycan influence. However, experimental evidence points to varying glycosylation patterns across Spike variants, which may influence the glycan-mediated functionalities described here (Zheng et al. 2022, Baboo et al. 2023, Li et al. 2023). These effects are primarily attributed to glycan composition, which we did not evaluate in this study. The gain of N-glycosylated sites in particular lineages (Baboo et al. 2023, Shajahan et al. 2023), such as at ASN354 (Liu et al. 2024), has also been reported. These additions, while having the potential to affect our conclusions, cannot be assessed within our methodology due to the absence of structures with glycans bound to these sites of interest in our dataset. Additionally, the glycosylation patterns of Spike can be affected by the specific cell types in which it is expressed (Goh and Ng 2018). As these data are not readily available programmatically, it is possible that the patterns of glycan interactions observed could not represent those *in vivo*. However, to the extent that such an experimental artefact may be averaged out, the fact that we utilized approximately 1000 Spike proteins for the analysis of glycosylation may restrict the extent of this problem.

### 3.5 Enthalpic trade-off hypothesis: receptor-binding affinity and antibody recognition

The enthalpic trade-off hypothesis is based on the underlying assumption, as previously proposed (Teruel et al. 2021b, Bozdaganyan et al. 2022, Li et al. 2024), that mutations in the same surface of the Spike protein will affect the binding of all proteins that interact with this epitope. Specifically, we analysed antibody interactions at epitope 4, which shares its binding interface with the ACE2 receptor (Fig. 1d). Given the dataset composition, we evaluated the interplay between antibody and ACE2 binding for the Alpha, Beta, Gamma, Delta, BA.1, BA.2, and BA.4/BA.5 variants. We compared ACE2 and antibody binding using two approaches: a per-residue decomposition of binding affinity (Fig. 8a) and an analysis of total per-variant interactions (Fig. 8b).

For variants like Alpha and Beta, per-residue evaluations show that mutations in epitope 4 affect both ACE2 and antibodies targeting the RBM in similar ways, either increasing both interactions (N501Y) or decreasing both (K417N, E484K), aligning with the enthalpic trade-off hypothesis. For Gamma, the K417T mutation shows a more subtle immune escape effect, slightly increasing or decreasing binding affinity to antibodies, which may reflect the dataset limitations for Gamma (see Section 3.1). The only Delta mutation detected in interactions with both ACE2 and antibodies targeting epitope 4 occurs at position 478, showing no effects on ACE2 binding but variable effects on antibody recognition.

Omicron subclades carry many mutations in the RBM. In BA.1, the effects of mutations at positions 501, 484, and 417 resemble those in Beta, while new substitutions show milder effects. T478K causes a small decrease in antibody recognition and a slight increase in ACE2 binding, similar to Q493R (Fig. 8a). In the context of BA.1, Q498R also presents this effect, with a more prominent increase in ACE2 binding. S477N and G496S increase both ACE2 and antibody binding slightly. Y505H and G446S cause a minor increase in antibody binding and a decrease in ACE2 binding (Fig. 8a). In BA.2, T478K, Q498R, and S477N shift towards less favourable antibody binding (Fig. 8a). For BA.4/BA.5, Y505H and N501Y mutations show reduced antibody recognition, marking the first time N501Y does not strongly favour antibody binding (Fig. 8a). This suggests a possible epistatic effect for Omicron subclades, shifting the per-residue binding contribution of residues 501, 505, 477, and 478 towards decreased immune recognition. BA.4/BA.5 also introduces the F486V mutation, significantly reducing binding to both RBM-targeting antibodies and ACE2. In the full interaction analysis (Fig. 8b), BA.1 shows the most significant dual effect, increasing binding for both antibodies and ACE2, while BA.4/BA.5 decreases both interactions.

Considering all vectors of ACE2 interaction, we can search for the vectors of antibody interactions that most closely resemble interactions with the receptor. These are of antibodies P2B-1A1 (PDB 7CZP), Beta-29 (PDB 7PS2), and CTC-445.2 inhibitor (PDB 7KL9), which may increase the enthalpic trade-off observation and force a diminished ACE2-binding capacity for mutations selected for their immune escape effects. The binding similarity to ACE2 possibly makes these antibodies valuable in guiding the design of new therapeutic strategies.

In summary, our analysis shows a number of mutations supporting the enthalpic trade-off hypothesis, notably mutations N501Y, K417N, E484K/A, and F486V.

### 3.6 Entropic trade-off hypothesis: conformational dynamics and RBM interactions

Whereas the enthalpic trade-off hypothesis has been previously suggested, the combined analysis of interaction enthalpies and their effect on dynamics allows us to introduce the concept of entropic trade-off in the evolution of SARS-CoV-2, which may contribute to the evolution of any virus in which different conformational states of viral proteins are involved in the cell entry mechanism. The entropic trade-off hypothesis stems from the relationship between conformational dynamics and receptor/antibody binding (Fig. 8c and d). Studies have linked the closed conformation of the SARS-CoV-2 Spike protein to immune escape (Weissman et al. 2021), a phenomenon particularly relevant for variants like BA.1 and BA.2 (Calvaresi et al. 2023). These variants prioritize a closed conformation as a strategy to evade immune recognition, even at the cost of reduced ACE2-binding affinity due to decreased RBM exposure.

Conformation-specific antibody binding, especially for epitopes 3 and 4, warrants particular attention, as discussed earlier (Fig. 1f). Given the dataset composition, we evaluated the interplay between conformational dynamics and conformation-specific binding for Alpha, Beta, Delta, Kappa, BA.1, BA.2, and XBB.1 variants. The Omicron subclades (BA.1, BA.2, and the recombinant XBB.1) demonstrate a trend towards favouring the closed conformation, while the effects on antibody binding remain mild or even increase in the case of BA.1 (Fig. 8c). This observation suggests that immune escape in these variants stems from their conformational preference for the closed state, rather than a reduction in binding affinity at the interface. When analysing ACE2 binding and conformational dynamics, we observe increased ACE2-binding affinity in BA.1 and XBB.1, though effective binding may be compensated due to the lower occupancy of the open state.

## 4. Conclusion

The vast structural data on the SARS-CoV-2 Spike protein offer unprecedented opportunities for in-depth analysis using high-throughput computational methods. Our study shows how such an analysis can provide new insights into functional aspects of the Spike protein, particularly through a data-driven epitope classification system. By applying this approach, we categorized epitopes and developed a straightforward method for sorting interacting complexes within epitope clusters. We identified 14 distinct epitopes based on their conformational specificity, ACE2 binding, and glycan coverage, providing a detailed understanding of binding across different Spike protein domains. Our per-residue evaluations of antibody binding show high accuracy compared with experimentally determined immune escape scores for antibodies targeting the RBD, reinforcing confidence in our conclusions regarding less-studied domains also represented in our epitopes and providing a method for the evaluation of future strains or for different viruses. A longitudinal analysis of mutations within each epitope reveals that mutations in the NTD merit consideration for their potential effects on immune recognition in the latest variants. This analysis also highlights opportunities for antibody development and therapeutic alternatives targeting well-conserved epitopes. This new epitope classification and mapping facilitates a focused analysis of immune escape by narrowing it to the relevant subset of antibody structures associated with specific epitopes. For example, when studying ACE2 binding, by restricting the analysis to those antibody structures that have interactions with the RBM.

Our findings emphasize the importance of understanding how various functional aspects of the Spike protein interrelate, including its conformational dynamics, ACE2 receptor binding, antibody recognition, and glycan coating. While previous studies often evaluated these features separately, our comprehensive analysis reveals their interdependence and how they collectively influence the function and evolution of the Spike protein and consequently of the SARS-CoV-2 virus. By evaluating these aspects per variant, we connected them within our trade-off hypothesis and conducted a per-residue analysis to explain the success of specific substitutions as they first emerged. We also explored the epistatic effects of mutations in different variant contexts, such as the varying impacts on antibody recognition from mutations at positions 477, 478, 501, and 505 in various Omicron subclades. Regarding dynamical effects, we identified N501Y as a key mutation increasing open-state occupancy in early variants of concern, such as Alpha, Beta, and Gamma, while S375F in Omicron subclades indirectly facilitates immune escape by reducing open-state occupancy. While the conformational shift towards the closed state of Spike has been suggested as a mechanism for immune escape, we introduce a methodology based on biophysical concepts to quantify these distinct functional effects across mutations and variants. We hope that the now clearly defined concepts of enthalpic and entropic trade-offs will help characterize new mutations and understand the evolution of future variants of SARS-CoV-2 as well as other viruses. Additionally, structural evaluations highlight the role of glycosylation, particularly ASN343-linked glycans in mediating antibody recognition and ASN234-linked glycans in modulating dynamics.

Understanding these connections is essential for gaining deeper insights into the evolutionary pressures acting on the Spike protein. The methods and findings presented in this study lay a solid foundation for future research into the structural biology of the Spike protein, offering new avenues for exploring its role in the adaptability and pathogenicity of SARS-CoV-2. Additionally, the methodologies we introduce can be used for the analysis of other large structural datasets, a need that is becoming increasingly relevant with the growing number of available structures, both by the popularization of structural biology techniques for structure determination and the ongoing revolution in protein modelling.

## Supplementary Material

veaf027_Supp

## Data Availability

The data supporting the findings of this study are publicly available at https://github.com/nataliateruel/Comprehensive_review_Spike. This repository includes all PyMOL sessions used to generate the images, the raw data for interaction evaluations and dynamical assessments, the mutations per epitope per variant from the longitudinal studies, as well as Python scripts for classifying complexes among the 14 epitopes described.
