# Supplementary material for: Comprehensive analysis of SARS-CoV-2 Spike evolution: epitope classification and immune escape prediction
Source: Virus Evol. 2025 Jun 11;11(1):veaf027. doi: 10.1093/ve/veaf027 (PMC12159738; doi:10.1093/ve/veaf027)
Supplement: veaf027_Supp [file ResponseReviewers.pdf › suppl_data/ResponseReviewers.pdf]

Université de Montréal

Faculté de médecine
Département de pharmacologie et physiologie

18 of Mars, 2025

To the Editor

I would like to thank the reviewers and the editor for their thorough work and the reccomendation to publish the paper with minor corrections. I apologize for having taken so long to submit a revised version where the minor corrections were fully addressed. This delay came as a result of the first author having successfully finished her PhD and being in transition between jobs and countries of residence to take up a postdoctoral position. I am submitting today a fully revised version accounting for all reviewer requests. The reviewer requests and our detailed responses appear below, with the text written by the reviewers in blue. Together with the revised manuscript, I am also submitting a PDF of the revised manuscript with changed to the text highlighted.

I would like to thank you and the reviewers again for your work.

Kind regards,

Rafael Najmanovich, PhD.
Full Professor
Department of Pharmacology and Physiology

Response to reviewers

Reviewer: 1

Comments to the Author
Unprecedented efforts have been dedicated to isolating and characterizing anti-SARS-CoV-2 antibodies since the onset of the COVID-19 pandemic. The rapid evolution of SARS-CoV-2 has led to the emergence of numerous Spike protein variants and significant immune escape. In this study, the authors analyzed 1,560 published Spike protein structures, identifying 14 key epitopes. They developed a structure-based computational method to predict the escapability of specific mutations, showing strong correlation with DMS data. Additionally, the authors observed an enthalpic trade-off in Spike protein mutations, balancing antibody escape with reduced ACE2 affinity. However, since computational tools for predicting mutational binding loss already exist and the "trade-off" concept is widely accepted, the authors should further emphasize the novel contributions of this manuscript.

Thank you for your comments. The novelty of our work lies in the integrated evaluation of Spike protein mutations across multiple functional aspects, as well as the unprecedented scope of 1,560 analyzed structures. Finally, we introduce two trade-off hypotheses by combining the evaluations of these different functional aspects

C.P. 6128, succursale Centre-ville
Montréal QC H3C 3J7
Téléphone : +1 (514) 343-6345
Télécopieur : +1 (514) 343-2291
rafael.najmanovich@umontreal.ca
nrglab.github.io

Université de Montréal

Faculté de médecine
Département de pharmacologie et physiologie

Our findings build on the well accepted concept of the enthalpic trade-off, but extend it by providing a structure-based methodology to quantify the dual effects of mutations on both antibody escape and ACE2 binding. Moreover, we highlight the epistatic constraints influencing this trade-off, particularly in Omicron subclades, where mutations at residues 501, 505, 477, and 478 shift per-residue binding contributions toward decreased immune recognition, despite some being associated with enhanced ACE2 affinity (Page 23).

Beyond the enthalpic trade-off, we introduce the entropic trade-off hypothesis (section "Entropic trade-off hypothesis: Conformational dynamics and RBM interactions"), supported by the Vibrational Difference Score (VDS) metric for single mutations (Fig 5D) and Occupancy Calculations for variant-wide conformational shifts (Fig 7C,D). These metrics show changes in the patterns of favored conformational dynamics in the timeline of the pandemic, further refining our understanding of immune escape and functional adaptation.

These contributions are now more clearly stated in the abstract, that has been reformulated, as well as in the introduction.

Specific comments:
1. Page 2, line 21 "Numerous studies have employed different experimental and computational methodologies to identify epitopes,16–27…" It is not clear to me how epitopes were identified using computational methods.

To improve clarity, we have revised the text to explicitly describe how computational methods have been employed for epitope identification in the referenced studies. Specifically, some employ computational techniques for the evaluation of structures, based on the inspection of fewer structures and using techniques such as buried surface area calculations (Deshpande et al. (2021), Yuan et al. (2021)). Others, such as N'Guessan et al. (2023), employ sequence-based methodologies for epitope enrichment mapping.

2. It is unclear to me what the criteria were for choosing structures for the analysis in this study. On page 2, lines 48-49, the authors state, "we perform a comprehensive structural analysis of 1,560 Spike protein structures." Are all these structures full Spikes? It appears that X-ray structures with domains or peptides of the antigen must have been included, as evidenced by the use of PDB 7NAB, as shown in Fig. S2. However, the identification of "neighboring Spike chains" (page 7, line 38) suggests that all structures must be full Spikes. The authors also mention "crystallization artifacts" (page 7, line 37), but it is unclear what this means. Perhaps the authors could clarify these points.

C.P. 6128, succursale Centre-ville
Montréal QC H3C 3J7
Téléphone : +1 (514) 343-6345
Télécopieur : +1 (514) 343-2291
rafael.najmanovich@umontreal.ca
nrglab.github.io

The details about the structure selection are described in “Structure selection and modeling”, mentioning that the dataset is composed not only but also by full homotrimeric structures.

The only interactions within the database of structures that were not evaluated were those based on “crystallization artifacts”. Namely, cases in which asymmetric units present more than one Spike chain and their interacting antibodies in the same crystal but do not represent the actual biological assembly of the Spike protein.

3. Definition of epitope classes as shown in Fig 1B don't seem to correlate with Fig 1A. Epitope 2 doesn't seem to be on RBD but was shown as an RBD epitope in Fig 1A. Likewise, Epitopes 7 and 8 which don't seem to be on NTD. Additionally, the “C-terminal domain” (epitope 1) should be S2 stem helix?

Fig 1A shows the RDB label for the clusters related to epitopes 3, 4, and 5, and the NTD label for clusters related to epitopes 9, 10, 11, 12, and 13. We changed Fig 1A so the domain-related labels are more clear. S2 stem helix is the correct nomenclature for the domain that composes epitope 1. This has been corrected in the text.

4. In Fig. 2 legend, S2H97 should be classified as site V, not class 4. Site V represents many antibodies, but was not classified in this study. In Table 2, S2H97 was classified to epitope 3, which is distant to its actual location.

In the raw data for escape scores from processed_data/escape_data.csv, Greaney et al. 2022 (reference 48), S2H97 is classified as class 4. There are, however, publications classifying it as class 5, such as Wang et al. 2022. Given the inconsistence in classification in the literature, we removed the S2H97 comparison from Fig 2 and Table 2.

5. The number of interaction vectors used in this study is unclear. For example, the number of interaction vectors for epitope 1 is reported as 16 for the WT but 0 for all other variants (Fig. S4). However, since the sequence of epitope 1 is 100% conserved across all variants, shouldn't the number of interaction vectors remain consistent across variants?

It would be a possibility to classify the variants based on the sequences of each epitope – indeed epitope 1 is in a very well conserved domain, and we can consider therefore that the interactions we see for the WT epitope 1 would be consistent for other variants as well. The method according to which we evaluated the variants each structure represents, however, was based on the sequence of the entire structure, as mentioned in “Structure selection and modeling”.

C.P. 6128, succursale Centre-ville
Montréal QC H3C 3J7

Téléphone : +1 (514) 343-6345
Télécopieur : +1 (514) 343-2291

rafael.najmanovich@umontreal.ca
nrglab.github.io

Université de Montréal

Faculté de médecine
Département de pharmacologie et physiologie

Reviewer: 2

Comments to the Author
Teruel et al. conduct a structural bioinformatics analysis based on the wealth of SARS-CoV-2 structures gathered over the last five years. By processing >1600 structures through structural modeling and energetics algorithms followed by different post-processing analyses, the authors (1) identify clustered antibody epitopes with energetics of interaction that match with experimental mutagenesis data (but extend to epitopes for which such data is not available); (2) rank evolutionary change at each epitope over analysis of longitudinal SARS-CoV-2 genomic sequences; (3) predict effects of mutation on ACE2 binding in agreement with literature; (4) predict changes in RBD up/down occupancy, and (5) identify mutational tradeoffs for antibody versus ACE2 receptor binding. Each individual conclusion seems to relate more or less to things that we already know from the literature, as acknowledged by the authors based on the extensive literature referencing they use to validate their observations. Instead, the primary impact of this paper is unifying each of these different factors into one integrated study. I have some comments primarily on presentation but find this an interesting approach on a unique dataset that appears to perform well based on comparison with prior data.

Comments:

Abstract is not as clearly written as the main text. Some focus on improving clarity of this important paragraph would be helpful

The abstract has been improved to more clearly highlight the most important contributions of the paper.

Fig. 1: the use of just one legend for the plotting characters across several panels is quite confusing. For example, per the legend, is the red circle in fig. 1C something about ACE2 interactions, as red circle is annotated in the legend? I suspect not, but I'm also colorblind enough where telling if all of the colors in Fig. 1B are match the colors in Fig. 1C is hard to do (for example, I don't see the epitope 1 "hot pink" in Fig. 2C). There is enough space in Fig. 1C that perhaps annotations could be added to each cluster just as they are labeled in 1B?

The coloring for epitope 1 was indeed misleading for Fig 1B – this is now corrected. We also added the epitope labels to Fig 1C. Another correction was related to the similarities between the markers for the center of epitope 13 (dark orange circle) and ACE2 interactions (red circle). To overcome that, now ACE2 interactions are marked in red squares and the legend was broken down for each PCA plot.

C.P. 6128, succursale Centre-ville
Montréal QC H3C 3J7
Téléphone : +1 (514) 343-6345
Télécopieur : +1 (514) 343-2291
rafael.najmanovich@umontreal.ca
nrglab.github.io

In Fig. 1C, it seems there are many antibodies clustered in RBD epitopes that diverge much further from the clustered epitope prototypes compared to other epitopes outside the RBD. What does this indicate?

The number of vectors interacting in these epitopes is much larger than in the rest of the epitopes (Fig S2A), a difference that in itself can justify more variability in the exact position of binding. Beyond that, the method we used for the 2D representation we see in Fig 1C, Principal Component Analysis, finds the directions of maximal variance, so areas with a higher density of data points can show more spread along the principal components. However, these variations do not translate into drastic changes in 3D.

The primary papers from which deep mutational scanning data were used do not seem to be cited.

References 48 and 49, related to the DMS data used for the comparison, are cited in page 8: "Beyond establishing the equivalence with Barnes' classes, our evaluation demonstrates that per-residue contributions as calculated with Surfaces can predict DMS-based immune escape calculations. Specifically, experimental immune escape scores 48, 49 (ranging from 0 to 1) align with per-residue interactions (measured as pseudo-energy, where more negative values indicate stronger interactions)."

Fig. 3A (and 5D) legend: could be helpful to sort the VOC/Clade legend in chronological rather than alphabetical order

The legends of the two images have been changed to chronological order.
